# Evaluation of factors associated with the anxiety and depression of female infertility patients

**DOI:** 10.1186/1751-0759-5-15

**Published:** 2011-12-23

**Authors:** Mariko Ogawa, Kiyoshi Takamatsu, Fumi Horiguchi

**Affiliations:** 1 Department of Obstetrics and Gynecology, Tokyo Dental College Ichikawa General Hospital, Chiba, Japan

**Keywords:** Infertility, Anxiety, Mood disorder, Psychological test, Mental health

## Abstract

**Background:**

Because the primary aim of infertility treatment is to achieve pregnancy, mental health care during this treatment is often neglected. However, the inability to conceive children is stressful for couples throughout the world. Thus, the purpose of this study was to investigate factors related to the anxiety and depression of female infertility patients.

**Methods:**

Participants included 83 Japanese women who initially visited the Reproduction Center of the Tokyo Dental College Ichikawa General Hospital to undergo testing and receive infertility treatment between February and April 2008. We administered two psychological tests, the Self-rating Depression Scale (SDS) test and the Hospital Anxiety and Depression Scale (HADS) test. We then examined the association of the test results with age, pregnancy and delivery history, employment status, duration of infertility, infertility treatment history, and male infertility.

**Results:**

As patient age increased, total HADS and depression scores also increased. No correlation was observed between duration of infertility and SDS or HADS scores. Results were similar when the presence and absence of delivery history was compared. Patients who underwent infertility treatment were more likely to have high HADS depression scores compared to patients who had not undergone treatment. Additionally, patients whose husbands were infertile had significantly lower total HADS and anxiety scores than those whose husbands were not infertile.

**Conclusions:**

Age and male infertility are factors that influence the presence of anxiety and depression in female infertility patients.

## Background

Infertility is defined as the inability to conceive after having unprotected intercourse for one year. In many cases, couples realize they are infertile only after attempting to become pregnant for some time. A healthy husband and wife must acknowledge that they may be infertile. The rate of infertility is high, at approximately 10%, and in many cases the cause is unclear. An increasing number of patients ultimately require assisted reproductive technology (ART), which is accompanied by physical and financial hardships [1].

Mental stress, particularly anxiety and depression, resulting from infertility may be due to various factors, including uncertainty of the cause of infertility, uncertain treatment duration, financial stress, and pressure from others who know the couple. However, studies have identified no psychological pathologies associated with the mental health of infertile patients [2]. Thus, improving mental health outcomes for such patients has no direct bearing on improving their odds of achieving pregnancy [3]. Treatment for mental health problems has not been examined in such patients. However, some studies have reported that when the mental health of infertile patients and pregnant women is compared, the rates of anxiety and depression of the former are significantly higher [4]. While these occurrences are lower in infertile patients than in those with chronic pain syndromes or HIV, stress levels are similar to those of cancer or coronary heart disease patients [5]. Eliminating psychological stress may also be necessary for successful infertility treatment. [6].

Women appear to have a higher rate of infertility-related stress than men [7,8]. Studies have also found that greater than half of the women receiving infertility treatment feel that infertility is the "most stressful experience of their lives" [7,9].

Several reports are available on factors associated with the anxiety and/or depression of infertile woman. Domar *et al*. found that depression peaked during the third year of infertility [10]. Vogsten *et al*. determined that a negative pregnancy test after undergoing IVF is an independent risk factor for mood disorder in women [11]. Ramezanzadeh *et al*. found that anxiety and/or depression were observed more in housewives than outside employees [12].

In Japan, few studies have been conducted to assess factors affecting the mental health of infertile patients. A literature search found only four articles in English addressing this subject. In one study, Matsubayashi *et al*. conducted psychological tests of infertile women and healthy pregnant women. They found that infertile women had significantly higher mental stress scores [13]. The study also found that a contributing factor to the anxiety and depression of these women was the "lack of support from their husbands" [14]. Based on the analysis of psychological tests, Chiba *et al*. found that stress affects a woman's duration of infertility [15]. Akizuki *et al*. determined that positive and negative social interactions are experienced by infertile Japanese women within their social networks [16]. However, little is known regarding the mental health of infertile Japanese women.

Moreover, very few reports have been conducted on the psychological background of female patients whose husbands have been found to be infertile. Only one study was found. Nachtigall *et al*. determined that a man's response to infertility closely approximates that of a woman if the infertility has been attributed to a male factor, but differs considerably if a male factor is not found [17]. Therefore, it is beneficial to investigate factors that cause mental stress to female infertility patients.

To investigate factors leading to the anxiety, depression, and stress of patients receiving outpatient treatment, we conducted psychological tests during the initial patient visit to the infertility outpatient clinic and compared the results by stratifying factors such as age and duration of infertility. Additionally, we examined whether or not psychological differences exist for couples in which the husband was previously known to be infertile.

## Materials and methods

The outpatient Reproduction Center of the Tokyo Dental College Ichikawa General Hospital, specializing in infertility treatment, treats many patients suffering from male infertility. We conduct advanced infertility treatments such as *in vitro *fertilization-embryo transfer (IVF-ET), including intracytoplasmic sperm injections (ICSI). We also proactively treat male patients by offering male infertility outpatient treatment by urologists. Additionally, we offer artificial insemination using donor sperm (AID).

Participants included 83 women who initially visited the Reproduction Center of the Tokyo Dental Collage Ichikawa General Hospital to undergo testing and receive infertility treatment between February and April in 2008. We administered two psychological tests, the Self-rating Depression Scale (SDS) test and the Hospital Anxiety and Depression Scale (HADS) test, to each patient at their initial visit. After arriving at the reception desk, we obtained informed consent and patients completed the tests while in the outpatient reception area.

The SDS test consists of a 20 question depression scale developed by Zung [18]. The Japanese version of this test was published in 1983 by Sankyoubou [19]. The test is frequently used to diagnose patients with depression because it can be used for patients ranging from high school age to elderly patients. The test is simple, convenient, and takes only 5 min to complete. A higher score is a stronger indication that the patient has depression.

The self-administered HADS test consists of 14 survey questions. Seven of the questions relate to anxiety and the remaining seven questions relate to depression. Responses to individual questions as well as total score are evaluated. The HADS test was published in 1983 by Zigmond *et al*. [20] and has been in worldwide use since then. The Japanese version was translated and published by Kitamura *et al*. [21]. Subsequently, Kugaya *et al*. reported using the test to study mood disorders in Japanese cancer patients [22]. Many studies have been conducted to evaluate the usefulness of the HADS test for analyzing the mental health of female patients [23-25]. The test is minimally burdensome to patients because it contains only a few questions and is suitable for evaluating the anxiety and depression of patients with physical ailments.

In this study, we chose HADS as a brief screening tool for anxiety and/or depression, and SDS as a standard measure of depression. The SDS and HADS are frequently used in the field of psychosomatic medicine. Many studies have been conducted on the SDS and HADS, and these tests have already been evaluated through validation studies. Recently, Saito *et al*. used the SDS with State-Trait Anxiety Inventory (STAI) to develop and assess the Cognitive Appraisal Scale for Infertility (CASI) [26]. In contrast, by using HADS, we can assess anxiety and depression.

We statistically analyzed the questionnaire results to investigate the association between test scores and age, pregnancy and delivery history, employment status, duration of infertility, infertility treatment history, and male infertility. The *t*-test was used to determine statistical differences between pregnancy and delivery history, employment status, infertility treatment history, and male infertility. Differences in age and duration of infertility were analyzed using repeated measure analysis of variance (ANOVA), followed by Fisher's protected least significant difference (PLSD) post hoc analysis. All analyses were performed using StatView (Version 5). Values of *p *< 0.05 were considered significant.

## Results

All women replied to both psychological tests. Average age of the 83 participants was 34.5 ± 4.5 years and average duration of infertility was 3.3 ± 2.8 years. We obtained 79 valid responses (95.2%) to the SDS test and 83 valid responses to the HADS test (100%). The average score on the SDS test was 38.5 ± 6.5 points, indicating a normal range, although the values were somewhat elevated. The total HADS score was 10.3 ± 4.9 points, with a mean anxiety score of 5.7 ± 2.7 points and a mean depression score of 4.6 ± 2.9 points. SDS and HADS scores, evaluated by risk factor, are shown in Table 1.

**Table 1:** SDS and HADS test scores by risk factor

		SDS score (mean ± SD)	HADS score (mean ± SD)		
			
	n		Total HADS score	Anxiety score	Depression score
**Age (years)**					
< 30	10	39.8 ± 5.9	7.4 ± 4.6	4.6 ± 2.9	2.8 ± 2.3
30-34	23	38.1 ± 6.7	9.9 ± 4.2	5.8 ± 2.7	4.1 ± 2.6
35-39	37	38.8 ± 6.2	10.8 ± 4.2	5.6 ± 2.2	5.2 ± 2.6 #1
≥ 40	13	37.4 ± 7.3	12.7 ± 6.7	6.3 ± 3.6	6.4 ± 3.8 #1, #2
**Pregnancy history**					
Present	27	38.7 ± 7.7	9.8 ± 4.5	5.4 ± 2.7	4.4 ± 3.2
Absent	56	38.4 ± 5.9	10.5 ± 5.1	5.8 ± 2.8	4.8 ± 2.8
**Delivery history**					
Present	12	40.5 ± 9.0	9.7 ± 5.7	4.9 ± 2.9	4.8 ± 3.9
Absent	71	36.5 ± 6.0	10.4 ± 4.7	5.8 ± 2.7	4.6 ± 2.7
**Employment status**					
Present	50	37.8 ± 6.7	9.8 ± 5.1	5.7 ± 2.9	4.1 ± 2.9
Absent	33	39.5 ± 6.1	11.0 ± 4.4	5.6 ± 2.6	5.4 ± 2.9
**Duration of infertility (years)**					
< 2	28	38.5 ± 5.4	11.1 ± 4.2	6.1 ± 2.6	5.0 ± 2.8
2-4	26	38.4 ± 6.7	9.9 ± 4.6	5.8 ± 2.8	4.1 ± 2.5
> 4	29	38.4 ± 7.4	9.9 ± 5.7	5.1 ± 2.8	4.7 ± 3.3
**Infertility treatment history**					
Present	21	38.8 ± 5.4	11.3 ± 4.3	5.7 ± 2.4	5.6 ± 3.0
Absent	62	38.3 ± 6.8	9.9 ± 5.0	5.6 ± 2.9	4.3 ± 2.8
**Male infertility**					
Present	24	38.9 ± 7.2	8.5 ± 5.4	4.5 ± 2.9	4.0 ± 3.0
Absent	59	38.3 ± 6.2	11.0 ± 4.4*	6.1 ± 2.6*	4.9 ± 2.9

*: *p *< 0.05 (baseline: present)

#1: *p *< 0.05 (baseline: ages < 30), #2: *p *< 0.05 (baseline ages: 30-34)

### 1. Age

We divided the participants into four age groups: younger than 30, ages 30-34, ages 35-39, and age 40 and over. On the SDS test, no differences were observed according to age group. A significant difference in depression score was observed between patients in their twenties and those of the 35-39 and 40 and over age groups (*p *< 0.05) on the HADS test. As age increased, participants exhibited a greater tendency towards depression according to the test.

### 2. Pregnancy and delivery history

No significant difference was observed between the SDS and HADS tests between women with and without a history of pregnancy. Results were similar when the presence and absence of delivery history was compared.

### 3. Employment status

When we divided participants into those who were employed (including part-time jobs) and those who were unemployed, we observed no difference in total and anxiety scores on the SDS and HADS tests. However, the depression score on the HADS test was generally higher, although the difference was not significant (*p *= 0.07) among unemployed compared to employed participants.

### 4. Duration of infertility

Participants were divided into three groups based on the duration of infertility; less than 2 years, between 2 and 4 years, and 4 or more years. No significant difference was observed among the groups when scores from each psychological test were compared. Thus, there was no direct association between infertility duration and anxiety or depression indications.

### 5. Infertility treatment history

We compared HADS depression scores for participants with and without infertility treatment histories at other clinics. The group with a history of infertility treatment generally had higher values compared to those without such a history. This suggests that depression is more likely to occur in participants with a history of infertility treatment.

### 6. Male infertility

Participants were divided into two groups based on their awareness that the male partner was a factor in the couple's infertility at the time of the initial clinic visit. The 24 male infertility cases included 8 cases of azoospermia, 4 cases of oligozoospermia, 2 cases of asthenospermia, 7 cases of erectile dysfunction (ED), 1 case of testicular cancer, and 1 case with both oligozoospermia and ED.

Total HADS and anxiety scores were significantly lower for women who knew that their husbands were infertile compared to those who did not know (*p *< 0.05), suggesting that participants in the former group experienced less anxiety.

## Discussion

In this study, we analyzed the mental health of infertile women by focusing on two crucial factors: anxiety and depression.

We used the SDS and HADS tests to evaluate the psychological states of study participants. When the two tests were compared, the HADS test produced a higher percentage of valid answers.

Although the data suggest an increased likelihood of depression as the participants aged, the results were not clear. Additionally, pregnancy history, delivery history, and duration of infertility did not appear to have an effect on depression or anxiety. In a previous study, Domar *et al*. reported that anxiety levels are the highest in the second and third year of infertility and that these levels decrease after six years [10]. However, this was not observed in our study. Further evaluation is necessary to assess additional topics such as variations in anxiety and depression after participants initiate infertility treatment and to evaluate a larger number of participants.

Our results indicate that unemployed women had a greater tendency towards experiencing depression than did employed participants, according to the HADS test. In Japan, married people are often asked by their families and neighbors why they do not have children. Unemployed women are particularly vulnerable to such pressure. It has been reported that role satisfaction is associated with lower distress for employed women and those in relationships compared to childless women [27]. Additionally, patients who had previously undergone infertility treatment had a greater tendency towards depression according to the HADS test at their initial visit. These women may be affected by despondency or a sense of urgency over the failure of prior treatment.

We found that patients with knowledge of their male partner's infertility had lower anxiety scores on the HADS test than patients who did not have such knowledge. Generally, patients who visit clinics due to male infertility are seeking advanced infertility treatment such as ART or AID. Studies have found that IVF patients have a relatively high tendency to experience anxiety or depression [28]. Women undergoing IVF, compared to women who are fertile, are reported to be several times more likely to experience depression and anxiety [10]. Because anxiety is lower in female patients whose male partners are infertile, infertile women may internalize anxiety and factors that contribute to their infertility. Additionally, because women in Japan are often viewed as responsible when a couple is infertile, psychological stress is reduced in women whose male partners are infertile. Sheiner *et al*. compared working women in couples in which the woman was receiving infertility treatment versus those in which the male partner was receiving treatment. They reported that "infertile women had lower scores on listlessness (one subscale from a burnout scale) than women with infertile husbands" [29]. While this result does not correlate with results from our study, there may be national or other differences that require further investigation.

Our findings suggest that employment status and if it is the male or female in the couple who is infertile influences anxiety and depression in infertile female patients, and that this anxiety may result from concern regarding the causes of infertility.

A limitation of the present study is that we did not examine the prevalence of anxiety and depression according to these (what do you mean by these?) criteria. A structured interview needs to be conducted. Furthermore, because we relied on patients coming to only one center, the data may be biased.

For outpatient infertility treatment, it is vital to not only conduct general tests on patients but also to provide mental health care that matches the patient's background. Further studies are required to clarify variations in mental health by examining the condition of patients after they have completed general infertility tests and when their treatment becomes more intensive.

## Competing interests

The authors declare that they have no competing interests.

## Authors' contributions

MO designed the study and analyzed the data. KT and FH conceived the study and participated in its design and helped to draft the manuscript. All authors read and approved the final manuscript.
